# News and Coming Events

**Published:** 1908-04-25

**Authors:** 


### NEWS AND COMING EVENTS.

Dr. John Rutherford Skinner has been elected mayor of the ancient town of Winchelsea.

Dr. J. Ranken Lyle has been appointed the first Professor of Midwifery in the University of Durham.

As President of the London Hospital, Queen Alexandra has sent a donation of £100 in response to the Lord Mayor's appeal on behalf of that institution.

Mr. Cyril Maude has consented to lend the Playhouse for a theatrical performance in aid of the funds of the Seamen's Hospital Society (*Dreadnought*) on June 23.

Dr. Bushell Anningson, of Cambridge, and Dr. J. Spottiswoode Cameron, Medical Officer of Health, Leeds, have been added to the Council of the Royal Sanitary Institute.

The resolution to confer the freedom of West Ham on Lord Lister, who was born in the county borough, was carried unanimously at a meeting of the Town Council last week.

Mr. Chisholm Williams, F.R.C.S. Ed., M.R.C.S. Eng., has been appointed medical officer to the X-ray Department at St. John's Hospital for Diseases of the Skin, Leicester Square.

Mr. George Prentiss Shepherd has given a donation of £10[illegible]000 to the Royal Surrey County Hospital for the erec[illegible] and endowment of a convalescent home for patients o[illegible] hospital.

The Gibbs prize of £4,000 offered by the New York Academy of Medicine for the best original research work on disease of the kidneys has recently been awarded to Dr. Norman Ditman, of New York.

A special course of training for commissioned officers and professional men preparing for the examination for inspectors of meat and other foods, conducted by the Royal Sanitary Institute, will commence on Monday, April 27, at 7 p.m.

The German Society of Tropical Medicine has nominated Professors Nocht and Plehn as delegates to participate in the deliberations to be held in London in the summer or autumn with a view to the organisation of an International Society of Tropical Medicine.

His Majesty has been pleased to nominate Charles Sissmore Tomes, Esq., F.R.S., F.R.C.S., L.D.S., to be, for five years from the expiry of his present term of office, a member of the General Council of Medical Education and Registration of the United Kingdom.

The Honorary Secretaries of King Edward's Hospital Fund for London have received at the Bank of England from the executors of Mr. Samuel Lewis the sum of £18,750, being a seventh payment on account of the legacy of £250,000 bequeathed by Mr. Lewis to the Fund.

A mental hospital for the city of Cardiff, erected at a cost of £349,000, was opened on April 15. At present accommodation is provided for 750 patients. Cardiff now lodges out 650 patients in various asylums, and these are to be removed at once to the new institution. The chairman of the Hospital Committee (Mr. Morgan Thomas) stated that the buildings had been erected at a proportionate cost of about £10 less than some of the London asylums.

The Annual Court of Governors of the Eve[illegible] Hospital for Sick Children was held on Wednesday, April 22. At this meeting Mr. C. H. Fagge, M.S., F.R.C.S., was appointed a consulting surgeon to the hospital.

The Therapeutical and Pharmacological Section of the Royal Society of Medicine will meet in the Apothecaries' Hall, Blackfriars, E.C., on Tuesday, April 28, at 4.30 p.m., when Dr. Leonard Guthrie, F.R.C.P., will deliver "Remarks on Rheumatism and Chorea in Children."

On Thursday, April 30, Mr. William Bateson begins a course of three lectures at the Royal Institution on "Mendelian Heredity" (the Tyndall lectures). The Friday evening discourse on May 15 will be delivered by Dr. Herbert Timbrell Bulstrode on "The Past and Future of Tuberculosis."

Professor E. A. Minchin will give a course of twelve lectures on hæmoflagellates and allied organisms at the Lister Institute, Chelsea, at 5 p.m., on Tuesdays and Fridays during May and June, beginning on May 5. The lectures are open free to all members of the university and to all medical men or registered medical students.

Mrs. Mary Edith Pechey-Phipson, M.D., formerly chief physician of the Cama and Allbless Hospitals, Bombay, died on April 15 at Folkestone. After taking the M.D. degree at Berne in 1877, Mrs. Pechey-Phipson was admitted a licentiate of the Royal College of Physicians, Ireland, during the same year. She had been Vice-President of the Bombay branch of the Royal Asiatic Society, and was a Fellow of the University of Bombay and of the Royal Academy of Medicine of Ireland.

Dr. Joseph Walker, M.D., M.R.C.S., a former President of the Odontological Society, late of Grosvenor Street, died on April 13, in his eighty-third year. He was admitted a member of the Royal College of Surgeons, England, and a Licentiate in Dental Surgery in 1860, and he subsequently took the M.D. degree at St. Andrews. He had been lecturer in dental surgery at Westminster Hospital and assistant dental surgeon and lecturer on mechanical dentistry at the Dental Hospital of London.

The Liverpool Education Committee on April 15 again discussed the desirability of either placing the medical inspection of school-children under the care of a senior medical officer at a salary of £400, and responsible directly to the Committee through the Director of Education, or of placing the responsibility in the hands of the city medical officer of health. It was decided by sixteen votes to nine to appoint a senior medical officer independent of the city medical officer to carry out the duties of inspection.

The President of the Royal College of Surgeons of England has been informed that an unauthorised circular is being issued to members of the College regarding the admission of women to the membership and fellowship, and that in this circular it is suggested that members should disregard the request of the Council that the answers to the question in the official circular should be in the form of simply "Yes" or "No." The President hopes that members will not allow themselves to be misled by this unofficial circular, and that, with a view to avoiding confusion as regards the result of the poll, they will record their answers in the official balloting-card in accordance with the instructions for voting contained thereon.

Dr. F. H. Champneys has been re-elected Chairman of the Central Midwives Board, and Mr. H. G. Fordham has been re-elected Honorary Treasurer.

The honorary degree of Doctor of Laws has been conferred by the University of Aberdeen upon Professor W. D. Halliburton, M.D., F.R.S., and Colonel Johnson, C.B., M.D., R.A.M.C.

Mr. Christopher Martin, Surgeon to the Birmingham and Midland Hospital for Women, will deliver the Ingleby Lectures, in the University of Birmingham, on May 7 and 14, on the subject of "Myoma of the Uterus."

The fourth International Congress of Medical Electrology and Radiology will be held at Amsterdam from September 1 to 5 of this year. The office, to which all communications should be addressed, is at Vondelstraat 53, Amsterdam.

In connection with the Royal Ear Hospital, Soho, the tenth annual subscription ball takes place at Princes' Piccadilly, on Thursday, April 30. The Duchess of Leeds, the Duchess of Manchester, and the Duchess of Marlborough are among the patronesses.

The sixty-fourth annual festival of the Ragged School Union and Shaftesbury Society will be held in the Queen's Hall on Monday, May 4. The afternoon meeting, at 3 p.m., is arranged for the first time specially for young people, and H.R.H. the Princess of Wales has consented to be present. The evening meeting will begin at 6.30 p.m.

Under the will of the late Dr. Nathaniel Rogers, the Senate of the University of London this year offer a prize of £100, open for competition to all members of the medical profession in the United Kingdom, for the best essay or dissertation setting forth the results of original investigations made by the candidate on any medical pathological subject during the preceding two years.

At the Cavendish Rooms, Mortimer Street, will be held from April 28 to May 1 a nursing and midwifery conference. Lectures will be delivered, admission to which and to the conference will be free. An exhibition will be on view of all the latest scientific inventions and of improvements introduced for the advance of knowledge among midwives, with especial reference to the requirements of recent Acts of Parliament.

An influential committee has been formed for the purpose of establishing a memorial to the late Professor Annandale, Professor of Clinical Medicine in the University of Edinburgh. It is proposed that a fund should be raised for the purpose of awarding a gold medal annually to the best student in clinical surgery at the final examination, to be known as "The Annandale Gold Medal in Clinical Surgery." In addition to this, it is thought that a bust of the late professor should be placed in the lecture theatre of the Royal Infirmary, Edinburgh, so long associated with his teaching. As the late Professor Annandale identified himself so closely and for so many years with the athletic life of the University, it has been suggested that, if there should be any surplus after providing for these objects, it might be devoted to some memorial in connection with the athletic club. The committee, among whom are Sir William Turner and Mr. J. M. Cotterill, have appointed Mr. H. M. D. Watson, C.A., 13 Rutland Square, Edinburgh, to act as their Secretary and Treasurer, and those who desire to subscribe are requested to communicate with him as soon as possible.

The annual meeting of the Royal Eye Hospital, St. George's Circus, Southwark, will be held at the hospital on Wednesday, April 29 at 4 p.m.

A meeting of the Royal College of Surgeons in Ireland will be held on Tuesday, May 5, at 4.30 p.m., to elect Examiners. Graduates of any university which may be from time to time recognised by the college are eligible for election as examiners in the subjects of general education. All the other examiners must be Fellows or Licentiates of the college, or professors or lecturers in any school of medicine recognised by the college. Candidates must lodge their applications in writing with the Registrar, at the college, on or before Tuesday, April 28, at 10 a.m.

The annual provincial meeting of the British Balneological and Climatological Society will be held at Harrogate on Saturday, May 16, at 3.15 p.m., when the Fellows will meet at the Royal Baths, where they will be received by the Mayor and Bath officials. A reserved corridor carriage will leave King's Cross Station, London, at 10 a.m. Various papers, entertainments and excursions have been arranged. On Monday, May 18, a train will leave Harrogate at 10.10 a.m. for the return journey to London. Fellows may take their wives and daughters and are allowed to invite medical guests, who will all be permitted to dine at the Fellows' dinner, and their hotel expenses will be charged at the same rate as for the Fellows—viz., one guinea each from Saturday afternoon to Monday morning, including all meals excepting lunch on Sunday. The honorary secretaries are Dr. S. P. Sunderland, 11 Cavendish Place, London, W., and Dr. F. A. Mouillot, Elm House, Harrogate, who desire to learn as soon as possible the number of Fellows and guests likely to be present.

### FORTHCOMING MEDICAL BOOKS.

Messrs. Cassell and Company announce for publication on April 24 two medical books: the first, a volume upon "Electrical Treatment," by Wilfred Harris, M.D., F.R.C.P., the second, "Diseases of the Nervous System," by H. Campbell Thomson, M.D., F.R.C.P.

